# Understanding the effectiveness of psychosocial services for older adults’ mental health in China: a systematic review and meta-analysis

**DOI:** 10.1007/s00127-025-02945-w

**Published:** 2025-06-27

**Authors:** Jingyuan Liu, Chunwei Lyu, Crystal Kwan, Xi Lan, Jie Deng, Jinxuan Zhang

**Affiliations:** 1 Department of Applied Social Sciences, The Hong Kong Polytechnic University, Kowloon, Hong Kong, China; 2 School of Educational Studies, Universiti Sains Malaysia, Penang, Malaysia

**Keywords:** Mental health indicators, Chinese older adults, Psychosocial services, Meta-analysis, Effectiveness

## Abstract

**Objective:**

Given the rapid development of psychosocial interventions for older adults in China and the significant mental health impacts of the COVID-19 pandemic, it is crucial to evaluate psychosocial interventions' effectiveness in promoting mental health of China's older population. To address this need, a systematic review and meta-analysis were conducted.

**Methods:**

We conducted a comprehensive search across nine electronic databases and Google Scholar for controlled trial studies published between 2018 and 2023. A meta-analytic approach with random-effects models was employed, and moderator analyses explored variability in effect size estimates.

**Results:**

Thirty-one studies with 5,941 participants were included. Guided by the WHO's framework, mental health indicators were categorized as positive or negative. Positive indicators reflect better mental health with higher values, while negative indicators show worse mental health. Significant effects were noted for negative (g = -1.21, 95% CI: -1.44, 0.99) and positive (g = 0.68, 95% CI: 0.51, 0.84) mental health indicators, moderating by geographic region, intervention type, setting, and delivery modality.

**Conclusions:**

Psychosocial services could significantly benefit Chinese older adults' mental health. The moderator and subgroup analysis suggests that the most effective interventions involve mental health professionals and utilize multifaceted approaches. Additionally, the results indicate that intervention duration is an important consideration, as shorter-term programs in Hong Kong exhibited relatively smaller effects.

## Introduction

Population aging is a rapidly accelerating global phenomenon, presenting significant public health concerns [1, 2]. The World Health Organization [3] projects that the proportion of the world's population aged 60 and above will nearly double from 12 to 22% between 2015 and 2050. As home to the world's largest older adult population, China faces unique challenges [4]. In 2020, older adults accounted for 18.7% of China's 1.41 billion population, or 264 million individuals (Ning, 2021).

The mental health of this rapidly growing older cohort has become a pressing public health issue. These challenges are driven not only by physical health conditions, but also by social factors such as the loss of connections due to life events like bereavement, retirement, or elder abuse [5]. In China, mental health issues of older adults are significant. In 2017, Xu et al. found a 1-month prevalence of mental health problems at 14.3% and a lifetime prevalence at 24.2% among 3,325 Chinese older adults screened via the General Health Questionnaire-12 and clinically diagnosed using the Structured Clinical Interview for DSM-IV. Deng and Liu [6] analyzed 10,556 CLHLS participants (aged ≥ 65) cross-sectionally, finding 18.95% cognitive impairment prevalence—often comorbid with mental health issues among Chinese older adults [7].

Given the growing awareness and concern surrounding mental health issues among older adults in China, an increasing number of studies have focused on this important topic [8, 9], Kong et al., 2019). It is encouraging to see that various psychotherapeutic interventions (e.g., Cognitive Behavior Therapy [10]), alternative psychosocial interventions (e.g., music appreciation sessions [11], interventions utilizing virtual reality (VR) technologies [Barsasela et al., 2021]), and supportive interventions (e.g., case management [12], psychological education sessions [13]) have been implemented and examined in studies aiming to support Chinese older adults’ mental health. However, despite these attempts at psychosocial services and the significant progress of China’s mental health system in recent years, current understanding of their comparative effectiveness remains constrained by fragmented methodologies and heterogeneous outcome measures that prevent cross-study synthesis [14].

While existing systematic reviews have primarily focused on evaluating the effectiveness of psychosocial interventions for depression and anxiety in older adults [15], Kaur, 2014; [16], a more comprehensive understanding of these services remains elusive. Notably, the impact of psychosocial interventions on other common mental health concerns, such as mood distress (e.g., stress, loneliness), as well as positive mental health indicators (e.g., sense of happiness) among older populations, have not been systematically examined. The importance of including these indicators is grounded in the World Health Organization’s [17] holistic definition of mental health, which encompasses not only the absence of mental disorders but also the presence of positive psychological well-being. This approach aligns with the dual-factor model of mental health [18], which has increasingly informed recent public health efforts to enhance both symptom relief and positive functioning. Additionally, subclinical mental health concerns like chronic stress or loneliness are prevalent in older adults and significantly affect their quality of life [19], reinforcing the need for broader outcome measures. Further, the COVID-19 pandemic, which emerged in 2020, has significantly affected the mental health of older adults. Factors such as social isolation, fear of infection, and reduced access to healthcare have contributed to higher levels of depression, anxiety, and loneliness in this population [20, 21]. In response, remote psychosocial interventions like telehealth have been developed to help address these challenges [22], Giebel et al., 2022; [12]. Thus, an up-to-date, comprehensive review that incorporates these recent advancements is therefore warranted to provide a timely and holistic understanding of psychosocial services for older adults, particularly within the Chinese context where the provision of such services has rapidly evolved.

Guided by the World Health Organization’s [17] conceptualization of mental health and Keyes’ [18, 23] dual-factor model, we categorize mental health indicators into two broad types: positive and negative. The dual-factor model views mental health as comprising two related but distinct dimensions—mental illness (e.g., depression, anxiety, stress) and mental well-being (e.g., happiness, life satisfaction, emotional and social well-being). According to this model, the absence of mental illness does not necessarily equate to the presence of mental health. Therefore, both dimensions must be assessed to gain a complete picture of an individual’s mental state.

In line with this framework, we define negative indicators as those associated with psychological distress, where higher scores reflect poorer mental health (e.g., depression, loneliness). In contrast, positive indicators reflect aspects of flourishing and well-being, with higher scores indicating better mental health (e.g., happiness, life satisfaction). This dual-factor approach allows us to move beyond a traditional deficit-focused lens and provides a more holistic understanding of how psychosocial interventions affect both the reduction of symptoms and the promotion of well-being in older adults.

## Method

This review followed the PRISMA guidelines [24] and was registered with the International Prospective Register of Systematic Reviews (PROSPERO, registration number: CRD42024342384).

### Search strategy

To reflect both the expansion in scope and the timeliness of psychosocial interventions for Chinese older adults, the literature search was limited to studies published between 2018 and March 2023. This decision was guided by two primary considerations. First, we aimed to broaden the range of mental health outcomes beyond depression and anxiety, incorporating both positive and negative indicators. Second, we sought to provide an updated synthesis of recent interventions, particularly in light of substantial developments following the COVID-19 pandemic, which began in late 2019 and led to a surge in remote and innovative intervention modalities.

Given these dual objectives, the five-year window represents a strategic and manageable scope that balances comprehensiveness with feasibility. This approach aligns with PRISMA 2020 recommendations that encourage reviews to reflect the current state of rapidly evolving fields such as gerontology and mental health [25]. Earlier reviews have already synthesized evidence on pre-2018 interventions targeting depression and anxiety [15],Kaur, 2014; [16], while our review aims to build on this foundation by capturing the latest advancements and a broader array of mental health outcomes.

We employed three search strategies: searching nine electronic databases, conducting a manual search in Google Scholar, and reviewing the reference lists of included studies. The nine databases included two Chinese academic databases—China National Knowledge Infrastructure (CNKI) and WANFANG DATA—and seven English academic databases: Scopus, PubMed, Web of Science, MEDLINE, PsycINFO, PsycARTICLES, and Social Services Abstracts The search strategy included three sets of terms to capture studies related to Chinese populations, geriatric populations, and mental health indicators. The selection of relevant keywords, synonyms, and plural forms was guided by exploratory searches in databases like PubMed, as well as a review of published systematic reviews and meta-analyses focusing on mental health interventions for Chinese older adults (e.g., [16]). The search strategy incorporated terms related to Chinese populations, including ‘China,’ ‘Chinese’, ‘Hong Kong,’ ‘Macau,’ ‘Taiwan,’ and ‘Mainland.’ To capture studies involving older adults, keywords like ‘older,’ ‘elder,’ ‘senior,’ ‘aged,’ ‘geriatric,’ and ‘gerontology’ were used. Furthermore, mental health indicators were identified using terms such as ‘mental health,’ ‘mental disorders,’ ‘mental health indicators’ ‘psychosocial,’ ‘psychological,’ ‘distress,’ ‘depress,’ ‘anxiety,’ and ‘happiness’. The three sets of search terms were combined and searched in the title and abstract.

### Inclusion and exclusion criteria

Eligibility criteria for inclusion in the review mandated that studies employ a controlled trial design, with or without random assignment, to evaluate the efficacy of psychosocial interventions in promoting mental health outcomes among older adult populations. Within the context of this review, psychosocial interventions encompassed a broad range of non-pharmacological approaches, which included traditional structured talking psychotherapies such as cognitive-behavioral therapy, supportive psychosocial services (e.g., case management, psychological education sessions, distinct from traditional talk therapies), and alternative psychosocial modalities (e.g., music appreciation sessions) [16]. According to the United Nations definition (UNHCR, 2017) and considering China’s retirement age of 60 for men and 55 for women [26],'older adults'were defined as individuals aged 60 and above. Only studies where the sample exclusively consisted of participants aged 60 years or older were included. Studies were eligible if they measured mental health as either the primary or secondary outcome. For instance, a study examining the mediation of exercise on cognitive function through reduced depressive symptoms and improved sleep quality met the inclusion criteria [27, 28]. Given the frequent co-occurrence of negative mental health indicators and cognitive decline in older adults, studies that assessed both mental health and cognitive function outcomes were also included [7, 29]. Participants were restricted to older adults residing in China.

Studies were excluded from the review if they: (i) did not have a control or comparison group, (ii) lacked measures assessing mental health constructs (e.g., depression symptomology, anxiety manifestations), (iii) did not report sufficient statistical data for effect size estimation, (iv) were published in languages other than Chinese or English, (v) had no full-text available, or (vi) were not peer-reviewed.

### Screening and data extraction

The eligibility assessment and data extraction were carried out by a team of five independent researchers. Two researchers conducted the initial screening in a blinded manner, evaluating titles and abstracts to exclude clearly irrelevant studies. The remaining studies then underwent full-text examination by the same pairs of blinded researchers to determine adherence to the predetermined inclusion criteria. Any conflicting assessments (13%, 14/108) were resolved through discussion, and a third investigator was consulted if consensus could not be reached.

Data extraction was independently performed by two researchers and then cross-checked by a third to ensure accuracy. Data extraction for all studies included study design, sample size, participants’ characteristics, providers’ characteristics, and effect size data. Study design included several key aspects, whether randomization was employed or not, the characteristics of the control group, the intervention characteristics, and outcome measures. Participants’ characteristics included age, gender and marital status as well as other characteristics reported in studies. Providers’ characteristics included but were not limited to profession, education level, and clinical experience.

### Publication bias, risk of bias, and sensitivity

The potential for bias in the selected full-text articles was assessed independently by two reviewers utilizing the Cochrane Collaboration's risk of bias assessment tool, as delineated in the Cochrane Handbook for Systematic Reviews of Interventions (Higgins et al., 2019). In instances where the assessments of the two reviewers differed, discussions were held to reconcile the discrepancies and arrive at a consensus judgment.

Funnel plot and Egger’s test were employed in this review to visually depict the bias, whereby potential publication bias occurs when P < 0.05 for the Egger’s test [30]. Sensitivity analysis was used to assess the stability of the results [31].

### Data syntheses and analysis

The statistical analysis procedures adopted in this study adhered to the recommendations of Pigott [32]. Initially, we conducted a descriptive statistical analysis to summarize the key characteristics of all the studies encompassed in the review. Subsequently, effect sizes were computed for the reported outcomes of each included study. In particular, we calculated the standardized mean difference effect size statistic (Hedges’s g), adjusted for potential bias due to small sample size, using the Stata17 software package. To synthesize the findings across the included studies, a meta-analytic approach was employed, utilizing random-effects models with inverse variance weighting. The presence of moderate to substantial heterogeneity, ranging from 50 to 70%, was considered an adequate criterion for conducting moderator analyses to explore potential sources of heterogeneity in the effect sizes. Upon observing variability in the effect size estimates, we proceeded to conduct moderator analyses to investigate potential sources contributing to this heterogeneity. While moderator analysis enables the identification of statistically significant differences between the effect sizes of two subgroups, it does not provide insights into whether the individual effect sizes within each subgroup are statistically significant. Consequently, we complemented the moderator analysis with an examination of treatment effects among subgroups to enhance the clinical relevance and interpretation of the results.

## Results

### Search results

**Fig. 1:**
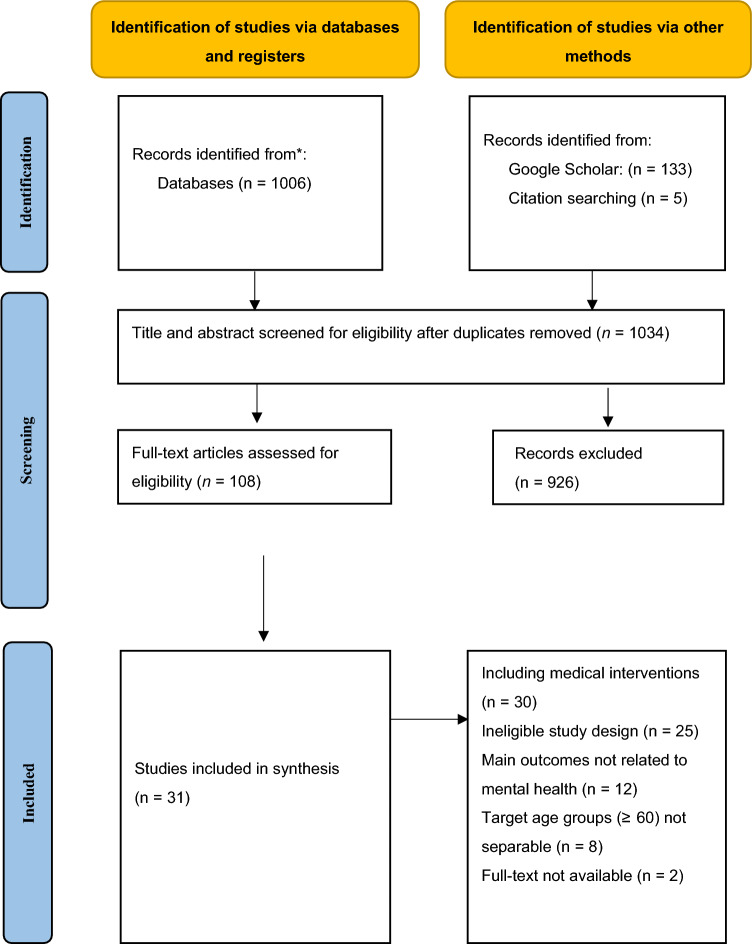
PRISMA diagram of search flow *Adapted from:* [25]

Figure 1 outlines the literature search and selection process. The initial pool comprised 1144 articles: 1006 identified through databases, 133 through manual searches on Google Scholar, and 5 from reference lists. After removing duplicates, 1034 articles were screened based on titles and abstracts, with 926 excluded for not meeting the inclusion criteria such as irrelevance to psychosocial interventions or non-target population. Full-text evaluation of the remaining 108 articles resulted in the exclusion of 77 studies that failed to meet the criteria (details in Fig. 1). Ultimately, 31 articles were included in the final review.

### Study characteristics

The review analyzed 31 studies with a combined sample size of 5,941 participants (see Table 1). Among these, 61.29% (19/31) included depression symptoms, 32.26% (10/31) included geriatric anxiety, and 22.58% (7/31) investigated other negative mental health indicators, including loneliness (3 studies), worry (2 studies), and mood or stress disorders (1 study). Five studies explored cognitive function, while 58.1% (18/31) assessed positive psychological outcomes such as happiness (n = 8), overall mental well-being (n = 2), self-esteem (n = 2), quality of life (n = 2), life satisfaction (n = 1), purpose of life (n = 1), self-efficacy (n = 1), and self-image (n = 1). Geographically, 61.29% (19/31) of the studies were conducted in mainland China, 19.35% (6/31) in Hong Kong, and 19.35% (6/31) in Taiwan. Most studies (74.19%, 23/31) used randomized controlled designs, while 25.80% (8/31) employed quasi-experimental designs. Regarding intervention types, 38.71% (12/31) tested therapeutic interventions, 35.48% (11/31) used alternative interventions (e.g., aerobic exercise, humor, VR, music), and 25.81% (8/31) implemented supportive interventions (e.g., case management, psychoeducation).

**Table 1:** Information of included studies

Article No	Author	Sample	Geo-area	Control	Demographic	Provider	Service characteristics	Outcome measures
1	An et al. [10]	T = 41 C = 22	Mainland	TAU	85.11 Y/O 31.7% male	Four interns with graduate-level education in social work	8 group cognitive behavior therapy (CBT) sessions 2 months	The Perceived Threat of Alzheimer's scale; The Alzheimer's Disease Knowledge Scale
2	Barsasella et al. [33]	T = 29 C = 31	Taiwan	NTC	T: 60–89 Y/O 13.79% male C: 60–94 years old 32.26% male	The Taipei Medical University (TMU) ageing center researchers	VR sessions for 15 min twice weekly Participants attended in groups, but VR individually conducted 6 weeks	EuroQol 5 dimensions 3 levels QoL; Mini version of the Chinese Happiness Inventory
3	Cai et al. [34]	T = 30 C = 30	Taiwan	TAU	79.8 Y/O 36.7% male	Mindfulness Guided experts	Mindfulness-Based Stress Reduction weekly session 9 weeks	State Anxiety Inventory
4	S. L. Chen et al. (8, 9)	T = 1232 C = 1133	Mainland	TAU	74.46 Y/O 33% male	Village primary care doctors with centrally located psychiatrists	Algorithm- driven treatment of depression and HTN Telephone consultation from centrally located psychiatrists 12 months	Hamilton Depression Rating Scale; Medical Outcomes Study Social Support Survey; Lubben Social Network Scale
5	Chen and Yang [35]	T = 32 C = 21	Mainland	NTC	T: 66.25 Y/O 56.43% male C: 65.23 Y/O 58.07% male	Four therapists	7 modules of CBT designed to address Generalized Anxiety Disorder (GAD) 12 sessions 3 months	Penn State Worry Questionnaire; Generalized Anxiety Disorder Severity Scale; GAD Questionnaire-IV; Beck Anxiety Inventory; Beck Depression Inventory; Intolerance of Uncertainty Scale; Why Worry-II; Cognitive Avoidance Scale
6	Chiang et al. [36]	T = 68 C = 62	Taiwan	TAU	T: 82.38 Y/O 42.2% male C: 80.63 Y/O 35.5% male	Social workers and researchers	The 3L-Mind-Training program, consisting of three modules: looking; listening; learning 6 weeks	The mini version of the Chinese Happiness Inventory (CHI-mini)
7	Chow et al. [37]	T = 68 C = 33	Hong Kong	Placebo	74.3 Y/O 18.4% male	Experienced bereavement counselors	DPBGI-C and LOBGI-C interventions weekly sessions 8 weeks	Inventory of Complicated Grief; Hospital Anxiety and Depression Scale; Loneliness Scale; Inventory of Social Support
8	Ho et al. [11]	T = 40 C = 33	Hong Kong	TAU	85.3 Y/O 30.0% male	Trainees from a local expressive arts therapy master program	A music intervention conducted with multi-sensory components 16 sessions 6 weeks	The Chinese version of the Neuropsychiatric Inventory The Visual Analog Mood Scale
9	Ho [38]	T = 18 C = 17	Mainland	TAU	T: 72.5 Y/O 61.1% male C: 72.5 Y/O 64.7% male	The primary care providers	Psychological nursing intervention: Targeted psychological counseling Cognitive interventions; Relaxation training 12 months	The Zung Self-Rating Anxiety Scale Zung Self-Rating Depression Scale
10	Jiang et al. [39]	T = 49 C = 43	Mainland	TAU	T: 70.4 Y/O 28.6% male C: 67.2 Y/O 25.6% male	Two psychological experts Four trained graduate students	Positive psychological intervention 12 sessions 7 months	Memorial University of Newfoundland’s Scale of Happiness
11	Man et al. [40]	T1 = 30 T2 = 34 C = 31	Hong Kong	NTC	T1: 82.1 Y/O 30.0% male T2: 84.06 Y/O 26.5% male C: 84.29 Y/O 16.1% male	Five Cantonese opera directors	Cantonese opera training program twice weekly, 10 sessions 5 weeks	Geriatric Depression Scale (GDS) The Cantonese version of the Mini-Mental State Examination The Positive and Negative Affect Scales
12	Lai et al. [41]	T = 22 C = 14	Hong Kong	NTC	T: 60.1 Y/O 22.7% male C: 59.8 Y/O 64.3% male	Professionals provide guidance on outdoor physical activities	Excursions to remote villages various outdoor activities six-day program	The 14-item Self-Image of Aging Scale for Chinese Elders The four-item self-report Subjective Happiness Scale
13	Lan et al. [42]	T = 31 C = 31	Mainland	TAU	83 Y/O 37.1% male	A registered nurse, skilled in guiding the life review process	The structured life review intervention six sessions 6 weeks	Short Portable Mental Status Questionnaire; GDS–15; Rosenberg Self-Esteem Scale; Purpose in Life Test
14	Li et al. [43]	T = 30 C = 30	Mainland	NTC	T: 65.2 Y/O 40% male C: 65.5 Y/O 33.3% male	Six professional psychology instructors; six nursing graduate students	Chinese traditional festival activity-group reminiscence therapy (CTFA-GRT) a four-hour intervention session 8 sessions in total 8 months	The Perceived Stress Scale; The well-validated 20-item UCLA loneliness scale
15	Lee et al. [44]	T = 105 C = 104	Hong Kong	NTC	71.5 Y/O 16.3% male 50.7% married`	Social workers, nurse, preacher, researcher, program workers	Modified mindfulness-based stress reduction (mMBSR) 8 weeks	Chinese Short Warwick-Edinburgh Mental Well-being Scale; GDS; Seven-item Pittsburgh Sleep Quality Index
16	Li [45]	T = 43 C = 43	Mainland	TAU	T: 76.2 Y/O 46.5% male C:76.3 Y/O 48.9% male	Nurses	Comprehensive nursing interventions: Health education; Dietary care; Psychological care; Exercise guidance 19 months	The Zung Self-Rating Anxiety Scale Zung Self-Rating Depression Scale
17	Luo et al. [46]	T = 24 C = 22	Mainland	NTC	T: 72.5 Y/O 79.2% male C: 74.6 Y/O 68.2% male	Maters-level social work research assistants	Face to face life review sessions participated by 4 to 6 participants 3 months	The 9-item scale Quality of Life Scale
18	Ma et al. [47]	T = 55 C = 58	Mainland	TAU	69 Y/O 69.0% male	Two professional trainers with Tai chi certification	Tai chi classes were offered twice a week in 8groups Each session lasted for 90 min 5 weeks	Social support rating scale; Center for epidemiologic studies depression scale
19	Peng et al. [13]	T = 54 C = 59	Mainland	TAU	T: 68.2 Y/O 37.0% male C: 68.8 Y/O 49.2% male	The designer controls and executes the cognitive training	Cognitive training (including memory training, attention training, and calculation training) 12 sessions 6 months	Beijing version of the Montreal Cognitive Assessment
20	Shuai and Liu [48]	T = 25 C = 25	Mainland	TAU	T: 69.7 Y/O 52% male C: 69.5 Y/O 56% male	A mental health rehabilitation team, composed of a team leader, clinical physician, social worker, and nurse	Mental health assessments, establishing care records; Regular group activities (reminiscence therapy); Regular health education sessions 12 months	The Zung Self-Rating Anxiety Scale; Zung Self-Rating Depression Scale; General self-efficacy scale
21	D. Song and D. S. F. Yu (2019)	T = 60 C = 60	Mainland	Placebo	75.78 Y/O 25% male	A nurse academician a physiotherapist exercise physiologists	Aerobic stepping exercise intervention Three group training sessions per week 4 months	GDS Pittsburgh Sleep Quality Index
22	Tsai et al. [49]	T = 32 C = 30	Taiwan	NTC	75.2 Y/O 33.9% male	The LINE app was the independent or staff-assisted platform used by participants	The smartphone-based videoconferencing program Once a week 6 months	The revised 10-item UCLA Loneliness Scale; GDS The Taiwanese version of the short-form health survey
23	Teng et al. [50]	T = 29 C = 26	Taiwan	TAU	T: 79.1 Y/O 37.9% male C: 74.6 Y/O 57.7% male	A social worker, scholar, as well as a psychologist trained in art therapy groups	Expressive therapy continuum (ETC) and media dimension variables (MDVS) Once a week 12 weeks	GDS Short Form
24	Wang et al. [51]	T = 36 C = 36	Mainland	NTC	68.6 Y/O 45.8% male	Four nurses with certification of Level II national psychological Counsellors	Psychological Self-help Intervention (P-PSI) 1 month	The Chinese Mental Health Scale
25	Wang et al. [52]	T = 33 C = 33	Mainland	NTC	81.08 Y/O 28.79% male	Two instructors with prior professional dance training	40 min dance sessions 3 weekly sessions 12 weeks	Montreal Cognitive Assessment; Mini-mental State Examination GDS-15
26	A. K. C. Wong et al. [12]	T = 34 C = 34	Hong Kong	Placebo	71.8 Y/O 17.6% male	Nurses and social workers	Weekly case management utilizing Telecare group supported by social team 3 months	General Self-efficacy Scale GDS
27	Xie et al. [53]	T = 37 C = 36	Mainland	TAU	T: 72.0 Y/O 42.5% male C: 71.9 Y/O 40.0% male	Four nursing students	The modified behavioral activation treatment (MBAT) 8 weeks	GDS Beck Anxiety Inventory Oxford Happiness Questionnaire
28	Yao et al. [54]	T = 23 C = 25	Taiwan	NTC	76.22 Y/O 56.25% male	The researcher, certified in long-term care and ethics	Group reminiscence once a week 9 weeks	Short Portable Mental Status Questionnaire; GDS -Short Form; Life Satisfaction Scale
29	Zhan et al. [55]	T = 692 C = 739	Mainland	TAU	65.6 Y/O 53.5% male	Family doctors, psychological consultants, nurses, psychologists, physicians	Basic mental health services and a series of community services 12 months	Patient health questionnaire; anxiety disorder questionnaire; Well-being questionnaire; Quality of Life Index
30	Zhao et al. [56]	T = 37 C = 37	Mainland	NTC	78.4 Y/O 47.3% male	Nurse practitioners, psychologists and gerontologists	Humor intervention program once a week 8 weeks	GDS; Hamilton Anxiety Scale; The Memorial University of Newfoundland Scale of Happiness
31	Zhang [57]	T = 25 C = 25	Mainland	TAU	T: 71.2 Y/O 60% male C: 73.5 Y/O 56% male	Primary doctor and nurses	Fall prevention health knowledge training; Psychological intervention; Physical exercise 14 months	Anxiety Self-Rating Scale Depression Self-Rating Scale

C = Control Group; T = Treatment Group; NTC = No Treatment Control; TAU = Treatment as Usual; Y/O = Years Old

Interventions were delivered by various providers: mental health professionals (38.71%, 12/31), interdisciplinary teams (25.81%, 8/31), and nurses (19.35%, 6/31). A small number of studies involved other professionals, such as Cantonese opera directors, outdoor activity specialists, and physical education teachers (3 studies). One study used participant-led interventions, and another did not specify the service providers.

### Meta-analytic results

As shown in Table 2, a total of 23 studies were included for the meta-analyses, examining interventions for negative mental health indicators. These studies reported 36 effect sizes, and the overall treatment effect was found to be high (g = −1.21, 95% confidence interval [CI]: −1.44, −0.99, p < 0.01). As presented in Table 3, a total of 18 studies were identified, focusing on interventions aimed at improving positive mental health indicators. These studies reported 23 effect sizes, and the overall treatment effect was found to be moderate (g = 0.68, 95% confidence interval [CI]: 0.51, 0.84).

**Table 2:** Meta-analytic result of an overall effect (Negative Mental Health Indicators)

N/K	Heterogeneity analysis		Effect Model	Meta-Analysis	
	I^2^	P		g	95%CI
23/36	93.8%	< 0.001	Random-Effects Model	−1.21	(−1.44, −0.99)

N = Number of studies; K = Number of effect sizes; g = Effect Size (Estimation of the small sample size corrected Hedges'g statistic); 95%CI = 95% confidence interval

**Table 3:** Meta-analytic result of an overall effect (Positive Mental Health Indicators)

N/K	Heterogeneity analysis		Effect Model	Meta-Analysis	
	I^2^	P		g	95%CI
18/23	73.7%	< 0.001	Random-Effects Model	0.68	(0.51, 0.84)

N = Number of studies; K = Number of effect sizes; g = Effect Size (Estimation of the small sample size corrected Hedges'g statistic); 95%CI = 95% confidence interval

### Moderator and subgroup analysis

Table 4 and 5 present results of the moderator analysis of studies exploring negative mental health indicators (group 1) and positive mental health indicators (group 2), respectively. Geographic areas, intervention types, intervention settings and modality significantly moderated treatment effect of group 1. Similarly, geographic areas and intervention settings significantly moderate treatment effect of group 2.

**Table 4:** Results of meta-regression (Negative Mental Health Indicator)

Predictor	β	95%CI	SE	t	P
Study design	0.15	(−0.90, 1.20)	0.52	0.29	0.771
Primary outcome	0.36	(−0.23, 0.96)	0.29	1.25	0.221
Geographic	0.62*	(0.13, 1.10)	0.24	2.57	0.015
Intervention	0.89***	(0.46, 1.33)	0.22	4.09	< 0.001
Modality	0.86**	(0.29, 1.44)	0.28	3.06	0.004
Delivery format	1.31	(−0.10, 2.72)	0.69	1.88	0.068
Setting	0.88**	(0.26, 1.50)	0.30	2.90	0.007
Provider	0.33	(−0.05, 0.71)	0.19	1.72	0.095
Control Group	0.01	(−0.57, 0.58)	0.28	0.02	0.986

β = regression coefficient; CI = confidence interval; SE = Standard Error

**Table 5:** Results of meta-regression (Positive Mental Health Indicators)

Predictor	β	95%CI	SE	t	P
Study design	0.22	(−0.19, 0.62)	0.19	1.12	0.277
Primary outcome	0.13	(−0.11, 0.36)	0.11	1.12	0.273
Geographic	0.46***	(0.25, 0.66)	0.10	4.68	< 0.001
Intervention	0.12	(−0.13, 0.37)	0.12	0.98	0.339
Modality	0.19	(−0.12, 0.49)	0.15	1.26	0.221
Delivery format	0.05	(−0.62, 0.73)	0.32	0.16	0.876
Setting	0.39*	(0.01, 0.77)	0.19	2.09	0.049
Provider	0.15	(−0.02, 0.31)	0.08	1.84	0.080
Control Group	0.07	(−0.13, 0.28)	0.10	0.74	0.0468

β = regression coefficient; CI = confidence interval; SE = Standard Error

Table 6 revealed the results of subsequent subgroup analysis. Within group 1, effect size estimates derived from studies conducted in mainland China, Taiwan, Hongkong, using therapeutic, supportive, and alternative interventions, and including small group, and individual interventions were statistically significant. In-person and mixed interventions were statistically significant. Interventions provided by mental health professions, interdisciplinary team and nurses were statistically significant. Interventions conducted in communities, hospitals, senior care homes were statistically significant. Table 7 showed that, for group 2, except for the effect size estimates from the study that utilized mixed-modality interventions, all effect sizes of various subgroups were statistically significant.

**Table 6:** Subgroup analysis of interventions’ effect (negative indicators)

Subgroup	N/K	I^2^	g (df)	95%CI	P
Study design					
RCT	18/29	94.1%	− 1.23 (28)	(− 1.47, − 0.99)	< 0.001
NRCT	5/7	92.6%	− 1.15 (6)	(− 1.93, − 0.38)	0.004
Primary outcome					
Anxiety	10/10	93.6%	− 1.54 (9)	(− 2.08, − 0.99)	< 0.001
Depression	19/19	94.5%	− 1.22 (18)	(− 1.53, − 0.91)	< 0.001
Other	6/7	86.0%	− 0.81 (6)	(− 1.32, − 0.30)	0.002
Geographic					
Mainland	15/25	94.0%	− 1.48 (24)	(− 1.74, − 1.22)	< 0.001
Hong Kong	4/6	0.0%	− 0.31 (5)	(− 0.46, − 0.16)	< 0.001
Taiwan	4/5	92.6%	− 1.03 (4)	(− 1.88, − 0.18)	0.018
Intervention					
TI	9/15	90.7%	− 0.88 (14)	(− 1.19, − 0.57)	< 0.001
SI	6/11	96.6%	− 2.41 (10)	(− 2.95, − 1.86)	< 0.001
II	8/10	87.6%	− 0.65 (9)	(− 1.06, − 0.24)	0.002
Modality					
Small groups	13/20	82.5%	− 0.84 (19)	(− 1.09, − 0.59)	< 0.001
Individual	8/13	95.7%	− 1.53 (12)	(− 1.88, − 1.19)	< 0.001
Mixed modality	2/3	98.1%	− 2.96 (2)^b^	(− 6.26, 0.34)	0.079
Delivery format					
In-person	20/32	93.4%	− 1.33 (31)	(− 1.59, − 1.07)	< 0.001
Tele Heath	2/3	0.0%	− 0.09 (2)^b^	(− 0.37, 0.20)	0.542
Mixed	1/1	N/A	− 1.37 (0)^a^	(− 1.44, − 0.99)	< 0.001
Setting					
Community-based	12/21	93.9%	− 1.07 (20)	(− 1.32, − 0.82)	< 0.001
Senior care homes	8/10	88.3%	− 0.87 (9)	(− 1.33, − 0.40)	< 0.001
Hospital	2/4	71.5%	− 3.17 (3)^a^	(− 3.86, − 2.48)	< 0.001
Elder’s home	1/1	N/A	− 0.20 (0)^b^	(− 0.67, 0.28)	0.422
Provider					
MHP	7/13	90.2%	− 1.03 (12)	(− 1.31, − 0.76)	< 0.001
Interdisciplinary team	8/10	95.7%	− 1.78 (9)	(− 2.37, − 1.18)	< 0.001
Not Mention/other	2/3	91.4%	− 1.43 (2)^b^	(− 2.74, − 0.12)	0.033
Nurse	5/8	94.9%	− 1.25 (7)	(− 2.02, − 0.48)	0.002
Independently	1/2	0.0%	− 0.03 (1)^b^	(− 0.38, 0.32)	0.869
Control group					
NTC	6/11	81.8%	− 0.68 (10)	(− 1.38, − 0.37)	0.001
Placebo	3/5	0.0%	− 0.36 (4)	(− 0.53, − 0.19)	< 0.001
TAU	14/20	94.7%	− 1.75 (19)	(− 2.04, − 1.45)	< 0.001

N = Number of studies; K = Number of effect sizes; g = Effect Size (Estimation of the small sample size corrected Hedges'g statistic); df = degrees of freedom, a lower P value of 0.01 should be adopted if df < 4; ^a^df < 4, but still considered statistically

**Table 7:** Subgroup analysis of intervention’s effect (Positive Psychological indicators)

Subgroup	N/K	I^2^	g (df)	95%CI	P
Study design					
RCT	12/15	69.2%	0.84 (7)	(0.46, 1.21)	< 0.001
NRCT	6/8	76.3%	0.61 (14)	(0.43, 0.80)	< 0.001
Primary outcome					
Happiness	8/8	77.9%	0.66 (7)	(0.40, 0.92)	< 0.001
Cognitive function	5/5	80.6%	0.53 (4)	(0.14, 0.92)	0.008
Other	8/10	65.7%	0.79 (9)	(0.50, 1.08)	< 0.001
Geographic					
Mainland	10/12	12.2%	0.69 (11)	(0.57, 0.82)	< 0.001
Hong Kong	4/7	25.7%	0.27 (6)	(0.09, 0.44)	0.003
Taiwan	4/4	76.6%	1.28 (3)^a^	(0.72, 1.83)	< 0.001
Intervention					
TI	5/7	79.1%	0.54 (6)	(0.22, 0.87)	0.001
SI	5/5	0.0%	0.75 (4)	(0.65, 0.85)	< 0.001
II	8/11	71.7%	0.74 (10)	(0.46, 1.02)	< 0.001
Modality					
Small groups	11/15	67.5%	0.78 (14)	(0.56, 1.00)	< 0.001
Individual	6/6	0.0%	0.74 (5)	(0.64, 0.83)	< 0.001
Mixed modality	1/2	0.0%	0.11 (1)^b^	(−0.08, 0.31)	0.252
Delivery format					
In-person	16/21	75.7%	0.67 (20)	(0.50, 0.85)	< 0.001
Tele Heath	2/2	23.2%	0.73 (1)^a^	(0.33, 1.12)	< 0.001
Setting					
Community-based	10/14	70.4%	0.55 (13)	(0.37, 0.73)	< 0.001
Senior care homes	6/7	79.6%	0.97 (6)	(0.53, 1.41)	< 0.001
Elder’s home	2/2	23.2%	0.73 (1)^a^	(0.33, 1.12)	< 0.001
Provider					
MHP	7/7	66.2%	0.92 (6)	(0.65, 1.18)	< 0.001
Interdisciplinary team	5/6	86.5%	0.57 (5)	(0.15, 0.99)	0.008
Nurse	4/6	49.8%	0.61 (5)	(0.35, 0.86)	< 0.001
Other providers	2/4	0.0%	0.48 (3)^a^	(0.16, 0.79)	0.003
Control Group					
NTC	9/12	62.9%	0.54 (11)	(0.31, 0.77)	< 0.001
Placebo	2/3	67.5%	0.68 (2)^a^	(0.27, 1.09)	0.001
TAU	7/8	72.5%	0.85 (7)	(0.60, 1.09)	< 0.001

N = Number of studies; K = Number of effect sizes; g = Effect Size (Estimation of the small sample size corrected Hedges'g statistic); df = degrees of freedom, a lower P value of 0.01 should be adopted if df < 4; ^a^df < 4, but still considered statistically

significant because its corresponding P < 0.01; ^b^df < 4, not considered statistically significant because its corresponding P > 0.01; 95% CI = 95% confidence interval.

### Sensitivity, publication bias and quality of studies

**Fig. 2:**
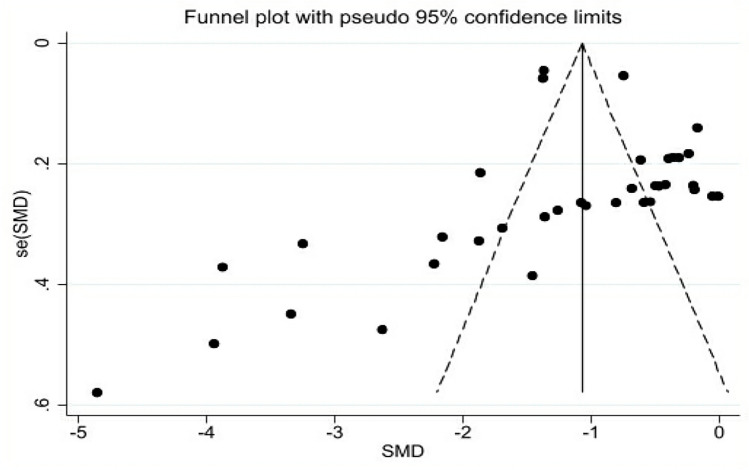
Publication bias for group 1

**Fig. 3:**
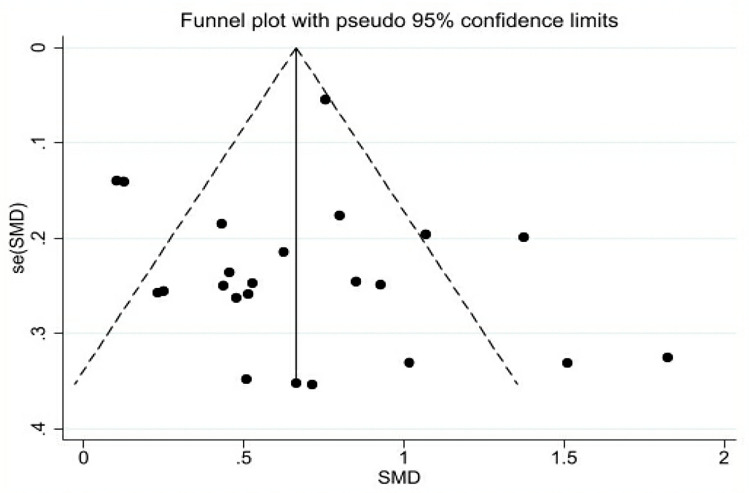
Publication bias for group2

As shown in Appendix A, in Fig. 2, for group 1, the Egger's test for publication bias yielded a p-value of 0.882, indicating that there is no evidence of publication bias as p > 0.05. Similarly, Fig. 3 showed that for group 2, the Egger's test for publication bias yielded a p-value of 0.929, indicating that there is no evidence of publication bias as p > 0.05.

**Fig. 4:**
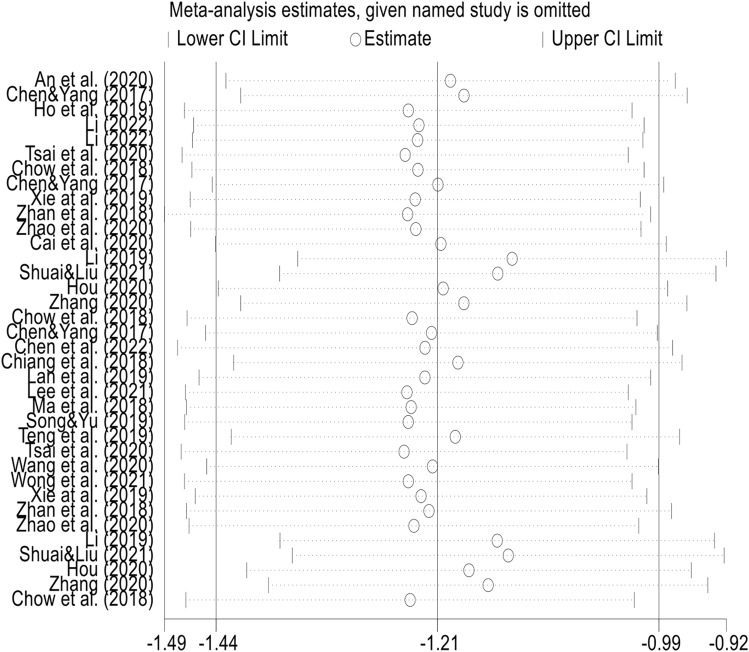
Sensitivity test for group 1

**Fig. 5:**
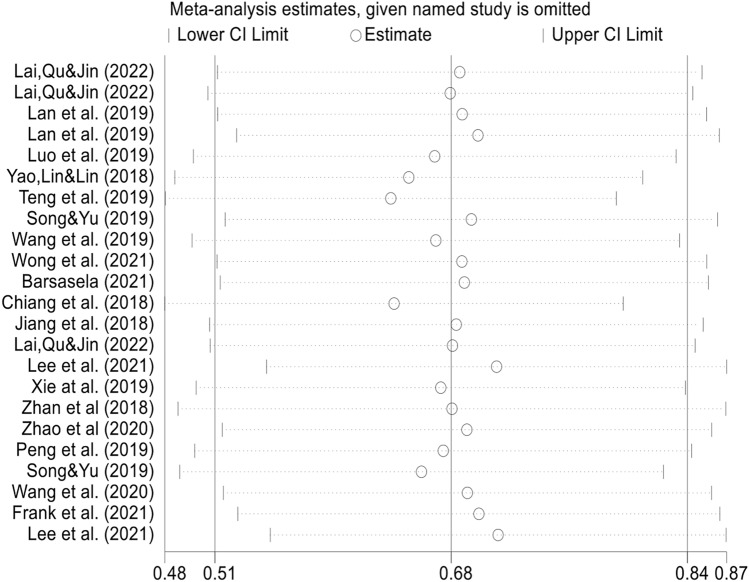
Sensitivity teat for group 2

Presented in Appendix B, Fig. 4 showed that after excluding a study that had the largest difference compared to other studies, the heterogeneity result was I^2^ = 93.3%, indicating a significant decrease in heterogeneity (originally, heterogeneity was I^2^ = 93.8%). This suggests that the influence of outliers on the results of this study is minimal, and the findings are relatively stable. In Fig. 5, for the group of positive psychological indicators, it is also shown in the figure that after excluding the study with the largest difference compared to other studies, the heterogeneity result was I^2^ = 70.3%, indicating a significant decrease in heterogeneity (originally, heterogeneity was I^2^ = 73.7%), which suggests that the findings of this study are relatively stable.

**Fig. 6:**
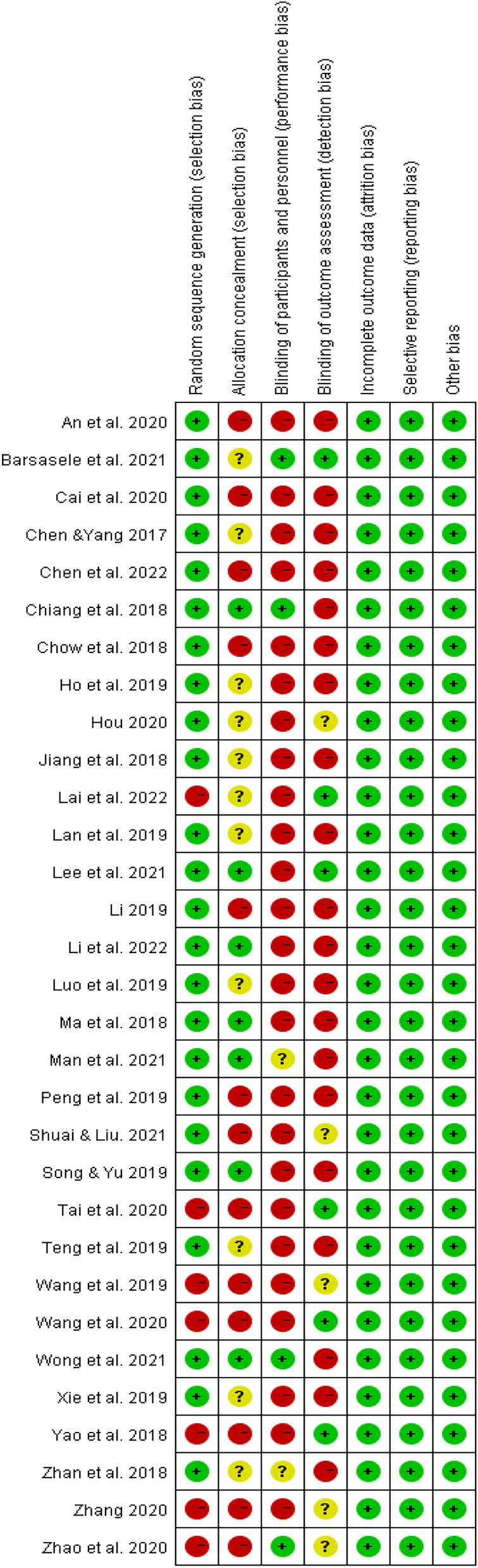
The individual risk of bias

In Appendix C, the findings from the quality assessments are depicted in Fig. 6. Overall, the included studies exhibited an average score of 4.26 out of 7, indicating a moderate level of study quality. Specifically, the studies demonstrated satisfactory adherence to criteria such as reporting random sequence generation (24/31), minimizing attrition bias (31/31), and avoiding reporting bias (31/31). However, the utilization of allocation concealment was found to be deficient in the included studies (7/31), as well as the appropriate implementation of blinding protocols for participants (4/31) and outcome assessments (6/31).

## Discussions

Mental health plays a vital role in the overall well-being of older adults. However, there is limited understanding regarding the effectiveness of psychosocial services specifically tailored for the Chinese older population. To address this gap, the present study conducted a comprehensive systematic review and meta-analysis of psycho-social services, encompassing interventions for both negative and positive mental health indicators among older adults.

The findings of this study revealed a significant and large treatment effect (g = −1.21, 95% CI: −1.44, −0.99, P = 0.000) of psycho-social services on the negative mental health indicators of older adults. Further, a significant and medium treatment effect (g = 0.68, 95% CI: 0.51, 0.84, P = 0.000) of psycho-social services was observed in improving positive mental health indicators among older adults. Compared to a previous review that focused on psychosocial services for geriatric depression and anxiety up until 2018, which reported a medium effect size (d = 0.577, 95% CI: 0.288, 0.867), results of this study are promising and suggest progress in the effectiveness of psychological services for older adults in China [16]. Based on the findings of this review, the favorable outcomes may be partly attributed to advancements in two key areas. First is the increased use of mental health professionals as intervention providers. Compared to the review conducted by Zhang et al. [16], which included only 3 studies (10%, 3/33) employing mental health professionals for delivering psychosocial interventions, this review reports a higher number of studies (63%, 19/30) that involve at least one mental health professional as a psychosocial intervention provider. This increase in the involvement of mental health professionals may contribute to more accurate assessments, validated approaches, comprehensive participant support, and ultimately enhance the effectiveness of interventions.

Second, the development and implementation of comprehensive and multifaceted supportive interventions may help to explain the overall improvement in services. The results indicate that both psychosocial services for negative mental health indicators and psychosocial services for positive mental health indicators showed greater effectiveness in terms of supportive interventions compared to therapeutic interventions and alternative interventions. Among the studies with supportive interventions, all seven used multifaceted approaches. These approaches incorporated various interventions such as therapeutic sessions, case management, psychological education sessions, and physical exercise. For instance, in a study focused on alleviating anxiety and depression symptoms in older adults with diabetes [45], supportive interventions were tailored to address multiple aspects. These included health education on diabetes, psychological care from specially trained nurses who listened to patients'concerns, exercise activities tailored to their conditions, and personalized dietary recommendations based on individual needs. Such multifaceted approach could provide participants with a holistic experience encompassing physical, psychological, and social support. The positive association between multifaceted supportive interventions and their effectiveness underscores the importance of acknowledging the interconnectedness of biological, psychological, social, and spiritual factors when addressing mental health issues in older adults [58]. Thus, future interventions targeting Chinese older adults may need to consider a comprehensive intervention approach that provide supports from various aspects.

Contrary to expectations, studies conducted in Hong Kong reported significantly smaller treatment effects compared to those from mainland China and Taiwan, despite Hong Kong's longer history of mental health professions and protocols. In group 1, the effect size for Hong Kong studies was g = −0.31, compared to g = −1.48 for mainland China and g = − 1.03 for Taiwan. In group 2, the effect sizes were g = 0.27 for Hong Kong, g = 0.69 for mainland China, and g = 1.28 for Taiwan. An analysis of the study characteristics in Hong Kong revealed a potential explanation—a preference for short-term interventions. The mean intervention duration across 6 Hong Kong studies was just 6.64 weeks, with 83% (5/6) using programs of 8 weeks or less, including one as brief as 5 days. In contrast, the mainland China studies averaged 74 weeks, with the majority (13/19) lasted for 3 months or longer. Similarly, Taiwan, with an average intervention duration of 11 weeks, and 67% (4/6) of studies lasted more than 8 weeks also reported a significantly larger effect size compared to Hong Kong. Existing evidence indicates that short-term psychosocial interventions may have limitations in addressing complex psychosocial issues and producing sustainable effects. For instance, a systematic review and Bayesian network meta-analysis conducted by Schweighoffer et al. [59] examined the efficacy of short-term psychological interventions in inpatient palliative care settings, and the findings highlighted the limitations of such interventions in effectively addressing anxiety, depression, and emotional distress experienced by patients receiving palliative care. Similarly, a study examining relationship distress before and after a brief couples'intervention highlighted the insufficiency of solely focusing on acceptance work to create lasting changes in relationship functioning, thus underscoring the limitations of short-term interventions in addressing complex relationship issues (Reyes et al., 2020). Therefore, careful consideration of intervention duration is crucial when designing further geriatric psychosocial services to avoid potential weakening of effects associated with short-term interventions. This finding is particularly important in contexts that are increasingly favoring efficiency, individual responsibility and market-driven approaches to social issues that favor shorter-term yet potentially less impactful services.

## Limitations

This systematic review and meta-analysis possess inherent limitations. It should be noted that due to limited degrees of freedom in few analyses, the statistical inferences drawn from those results may be inconclusive. But the use of advanced methods for meta-analysis and moderator analysis still provides valuable insights into the effectiveness of psychosocial interventions for the mental health of Chinese older adults. Despite rigorous independent and blinded data screening by two researchers, the possibility of human error cannot be entirely excluded.

Beyond the limitations associated with meta-analysis, the included studies lacked sufficient data to compare treatment effects between in-person and telehealth modalities. Given the growing prominence of telehealth, particularly during the COVID-19 pandemic, future research should prioritize evaluating the comparative effectiveness of telehealth and in-person psychosocial interventions for Chinese older adults.

## Data Availability

Data is provided within the manuscript.

## Appendix A. Publication bias

See Figs. 2 and 3.

## Appendix B. Sensitivity test

See Figs. 4, and 5.

## Appendix C. Quality assessment

See Fig. 6.
